# P-486. Current Trends in Hospitalization Among People Living with HIV at an Inner City Hospital in Newark, N.J

**DOI:** 10.1093/ofid/ofae631.685

**Published:** 2025-01-29

**Authors:** Monique A Prince, Modupeoluwa Owolabi, Wajeeha Aiman, Beatrice Attilus, Kelly Mbenga, Jihad Slim

**Affiliations:** New York Medical College at Saint Michael's Medical Center, Newark, New Jersey; New York Medical College at Saint Michael's Medical Center, Newark, New Jersey; Saint Michael's Medical Center, Bloomfield, New Jersey; St. George’s University School of Medicine, St.George’s, Grenada, Naples, Florida; St. George's University School of Medicine, True Blue, Saint George, Grenada; Saint Michael’s Medical Center, Newark, NJ, USA, Newark, New Jersey

## Abstract

**Background:**

As HIV disease is becoming a chronic outpatient condition, we were interested in understanding what is driving high-cost hospitalization in people living with HIV (PLWH) at our inner city hospital in Newark, New Jersey. We sought to examine the trends in admissions over the last two years.

**Table 1:**
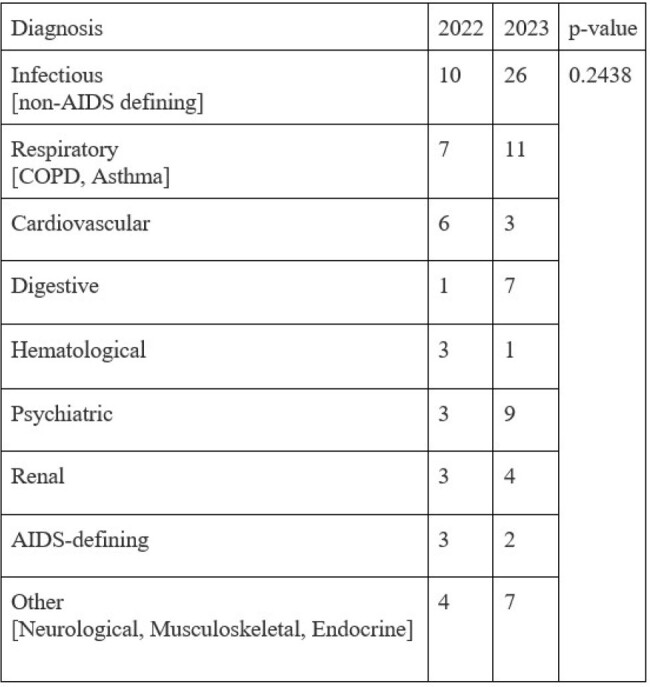
compares the causes of hospitalization between 2022 and 2023 [n = 110]

**Methods:**

A retrospective study was performed on electronic medical records of PLWH >18 years old admitted for at least 24 hours to the medical, intensive care, or psychiatry unit of Saint Michael's Medical Center from 2022-2023. The reason for hospitalization was determined to be the main diagnosis related to the symptoms on admission upon chart review. We calculated the standardized mortality ratios using observed deaths and expected deaths compared to mortality in the general inpatient population. We used R software to compare the causes of hospitalization between the two years using the Welch two-sample t-test.

**Figure 1:**
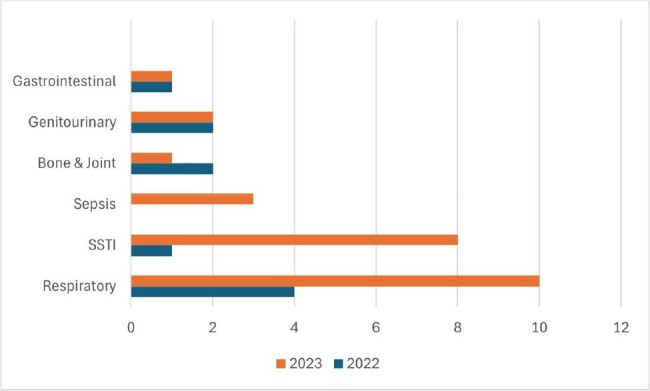
demonstrates the breakdown of causes by infectious diseases over the 2 years

**Results:**

We identified 110 HIV-positive adults admitted to the hospital. The mean age was 55.8 +/- 12.7 in 2022 and 53.6 +/- 16.3 in 2023. The causes of hospitalization are illustrated in Table 1, p=0.2438. Infections were the top cause of admissions with the most common infections from the respiratory system over two years as demonstrated in Figure 1. AIDS-defining infections were only 4.5% with a decreasing trend during the study period. Overall inpatient mortality in PLWH was 10% in 2022 and 7.14% in 2023. The mortality rate decreased from 4.06 (95% CI 2.20 - 6.91) in 2022 to 1.91 (95% CI 0.89 - 4.06) in 2023. The length of stay (LOS) among PLWH was 26% shorter than the LOS of the general inpatient population in 2022 (n = 4.475) and the same in 2023 (n = 5.34). The mean LOS in PLWH increased by 19.32% over the two years.

**Conclusion:**

Infections are the most common cause of hospitalization in PLWH with stable trends over the last two years. Average mortality rates and length of stay were lower in PLWH compared to the general hospital admissions.

**Disclosures:**

**Jihad Slim, MD, FACP**, AbbVie: Grant/Research Support|AbbVie: Honoraria|AbbVie: Speaker Bureau|Gilead Sciences, Inc.: Grant/Research Support|Gilead Sciences, Inc.: Honoraria|Gilead Sciences, Inc.: Speaker Bureau|Merck: Grant/Research Support|Merck: Honoraria|Merck: Speaker Bureau|Theratechnologies: Advisor/Consultant|Theratechnologies: Honoraria|ViiV Healthcare: Advisor/Consultant|ViiV Healthcare: Grant/Research Support|ViiV Healthcare: Speaker Bureau

